# The efficacy of intracolonic vancomycin for severe *Clostridium difficile* colitis: a case series

**DOI:** 10.1186/s12879-016-1657-1

**Published:** 2016-07-07

**Authors:** Christine M. Akamine, Michael B. Ing, Christian S. Jackson, Lawrence K. Loo

**Affiliations:** Department of Medicine, Loma Linda University School of Medicine, 11234 Anderson Street, Loma Linda, CA 92354 USA; Section of Infectious Disease, VA Loma Linda Healthcare System, 11201 Benton Street, Loma Linda, CA 92357 USA; Section of Gastroenterology, VA Loma Linda Healthcare System, 11201 Benton Street, Loma Linda, CA 92357 USA

**Keywords:** Intracolonic vancomycin, *Clostridium difficile*, Colitis, Mortality, Risk factors

## Abstract

**Background:**

*Clostridium difficile* infection (CDI) unresponsive to the standard treatments of metronidazole and oral vancomycin requires aggressive medical management and possible surgical intervention including colectomy. Intracolonic vancomycin therapy has been reported to be particularly promising in the setting of severe CDI in the presence of ileus. This is a descriptive case series exploring the effect of adjunctive intracolonic vancomycin therapy on the morbidity and mortality in patients with moderate to severe CDI.

**Methods:**

A retrospective chart review was conducted on 696 patients with CDI seen at a single institution. Each patient was assigned a severity score and 127 patients with moderate to severe CDI were identified. We describe the clinical presentation, risk factors and hospital course comparing those that received adjunctive intracolonic vancomycin to those that only received standard therapy.

**Results:**

The group that received adjunctive intracolonic vancomycin had higher rates of toxic megacolon, intensive care unit (ICU) admission, and colectomy, and yet maintained a similar mortality rate as the group that received only standard treatment.

**Conclusion:**

The intracolonic vancomycin group experienced more complications but showed a similar mortality rate to the standard therapy group, suggesting that intracolonic vancomycin may impart a protective effect. This study adds further evidence for the need of a randomized controlled study using intracolonic vancomycin as adjunctive therapy in patients presenting with severe CDI.

## Background

*Clostridium difficile* infection (CDI) is a major cause of antibiotic-associated diarrhea worldwide and the incidence of severe colitis is on the rise [1–4]. In 2011, it was estimated that CDI was responsible for almost a half million infections, approximately 29,000 deaths, and almost $5 billion in excess health care costs associated with acute care hospitalizations [4]. Severe CDI unresponsive to oral vancomycin and intravenous (IV) metronidazole therapy requires more aggressive medical management and possible surgical intervention [5]. Intracolonic vancomycin, or vancomycin administered per rectum, was initially described in the 1980s for use in cases of severe CDI [6–8]. In the case of paralytic ileus or patients unable to take oral medications, intracolonic vancomycin presented a promising alternative method for administering the antibiotic. Since then several groups have published reports of CDI treated with adjunctive intracolonic vancomycin with responses to therapy varying from 57 to 89 % [6, 9–12].

The Loma Linda Veterans Affairs (VA) Healthcare System is a 270 bed facility with approximately 120 acute care beds and is a major teaching affiliate of the Loma Linda University School of Medicine. Between October 2003 and March 2012, there were 696 patients admitted from the outpatient clinics and emergency room to the inpatient service with a confirmed diagnosis of CDI. We used a previously published severity score index [13] to identify patients with moderate to severe disease, and then compared the patients who received standard therapy with and without intracolonic vancomycin. The primary aim of our study was to determine whether or not adjunctive treatment with intracolonic vancomycin affected mortality in patients with moderate to severe CDI. We report a descriptive case series performed on 127 patients with moderate to severe CDI.

## Methods

### Study population and design

Between October 2003 and March 2012, 696 patients admitted to the inpatient service with a diagnosis of CDI were entered into an infection control surveillance database using a standardized information form that recorded patient demographics, clinical characteristics and risk factors, hospital course, and outcomes. The case definition of CDI included a patient with the clinical diagnosis of symptomatic diarrhea and one of the following laboratory or diagnostic criteria:

1. A positive enzyme immunoassay (EIA) for *Clostridium difficile* toxins A and B or
2. A positive glutamate dehydrogenase (GDH) assay with reflex confirmatory *Clostridium difficile* toxin detection by polymerase chain reaction (PCR) or
3. Endoscopic findings consistent with pseudomembranous colitis.

Using a previously validated clinical prediction instrument derived in a VA population, all patients were assigned a Severity Score Index based on the number of clinical and laboratory findings present within 48 h of the diagnosis of CDI (Table 1) [13, 14]. Severity scores were categorized as mild (0–3 findings), moderate (4–6 findings), or severe (7–9 findings) [14]. Of the 696 total patients, 569 patients with a severity score of 3 or less were determined to have mild CDI and were excluded from this study. This report focuses on the 127 remaining patients with moderate (108) to severe (19) CDI. A total of 26 patients received treatment that included intracolonic vancomycin while 101 patients received treatment without intracolonic vancomycin.

**Table 1:** Severity score index for *Clostridium difficile* infection [14]

Criteria	Points
Altered mental status (documented in the record)	1
Abdominal pain or distension (documented in the record)	1
Leukocytosis (WBC >20,000 cells/mm^3^) or leukopenia (<1500) or >10 % bands	1
Hypoalbuminemia (<2.5 mg/dL)	1
Ascites or colitis (documented by imaging)	1
Hypotension (MAP < 65 mm Hg)	1
Fever (≥101 ° F)	1
Tachycardia (≥110 bpm)	1
Admission or transfer to ICU	1

Interpretation: 0–3 is mild disease; 4–6 is moderate disease; 7–9 is severe disease

*WBC* white blood cells, *MAP* mean arterial pressure, *ICU* intensive care unit

### Data collection and patient outcomes

Demographic information included age, gender, and dates of hospital admission. Antibiotics and other medications taken 8 weeks prior to the diagnosis of CDI were recorded. Clinical symptoms and risk factors such as previous CDI within the past 12 months, ICU admission or transfer, presence of comorbid medical conditions, and recent vancomycin resistant enterococcus (VRE) or methicillin resistant *Staphylococcus aureus* (MRSA) infection and/or colonization were recorded. Laboratory data including a complete blood cell count (CBC), albumin, blood urea nitrogen (BUN), creatinine, albumin, and coagulation studies (PT, PTT, INR) were collected within 72 h of the diagnosis of CDI.

Charts of all patients enrolled in the study were reviewed for at least 90 days after diagnosis of CDI in order to determine outcomes. Outcomes were categorized as ICU admission, development of toxic megacolon, colectomy, and/or death.

### Statistical analysis

Differences between groups were analyzed using chi-square, Fisher’s exact and unpaired t-tests as approprite. Because of the exploratory nature of the study and multiple hypotheses testing, a *p*-value ≤ 0.01, rather than a *p* ≤ 0.05, was chosen to determine statistical significance [15].

## Results

### Baseline demographics, clinical characteristics and risk factors

Demographics, baseline characteristics, and risk factors of the patients in the study are summarized in Table 2. The majority of the patients were male (84.3 %), and the mean age was 68.5 years. The group that received intracolonic vancomycin had higher severity score indices on average compared to those who did not receive intracolonic vancomycin, although this did not meet the predetermined significance level (*p* = 0.03). The two groups had similar frequencies of major comorbidities and admission medications. Patients who received intracolonic vancomycin were more likely to have had a history of CDI prior to hospitalization, although this difference was not statistically significant (*p* = 0.06).

**Table 2:** Comparison of *Clostridium difficile* infected patients’ presentations between those who were treated with adjunctive intracolonic vancomycin and those who received therapy without intracolonic vancomycin

	Therapy with adjunctive intracolonic vancomycin No. (%) or average ± s.d.	Therapy without intracolonic vancomycin No. (%) or average ± s.d.	*P* value*
Total Number	26 (20.5 %)	101 (79.5 %)	
Severe Disease	7 (26.9 %)	12 (11.9 %)	0.11
Moderate Disease	19 (73.1 %)	89 (88.1 %)	0.70
Age (years + SD)	67 ± 8	70 ± 13	0.31
Male Sex	25 (96.2 %)	82 (81.2 %)	0.07
Average Severity Score Index for Total Group	5.69 ± 1.4	5.02 ± 1.0	0.03
Severe Disease	7.58 ± 0.98	7.08 ± 0.29	0.24
Moderate Disease	5.00 + 0.75	4.74 + 0.75	0.17
Past Medical History			
Recent surgery within last 6 weeks	9 (34.6 %)	25 (24.7 %)	0.31
History of CDI within the past 12 months	7 (26.9 %)	11 (10.9 %)	0.06
Comorbidities			
Diabetes mellitus	13 (50.0 %)	34 (33.7 %)	0.12
Coronary artery disease	9 (34.7 %)	26 (25.7 %)	0.37
Congestive heart failure	7 (15.4 %)	18 (17.8 %)	0.30
COPD	9 (34.6 %)	25 (24.8 %)	0.31
Malignancy	8 (20.7 %)	30 (29.7 %)	0.92
Cirrhosis	1 (3.8 %)	5 (5.0 %)	1.00
Admission Medications			
Proton pump inhibitors	13 (68.4 %)	62 (57.4 %)	0.45
Aspirin	8 (42.1 %)	36 (34.0 %)	0.46
H2 blockers	4 (21.1 %)	35 (32.4 %)	0.42
Corticosteroids	3 (15.8 %)	33 (30.1 %)	0.27
Laboratory Values within the first 72 h of the diagnosis of CDI			
WBC (cells/mm^3^)	27.2 ± 14.9	24.1 ± 16.9	0.36
Hgb (g/L)	10.1 + 1.9	10.0 ± 3.1	0.84
Albumin (g/dL)	1.7 ± 0.5	1.8 ± 0.5	0.55
BUN (mg/dL)	46.4 ± 28.2	42.3 ± 33.0	0.51
Creatinine (mg/dL)	2.2 ± 1.8	2.2 ± 1.8	0.97
Prior antibiotics within 8 weeks of diagnosis of CDI	24 (92.3 %)	94 (93.1 %)	0.76

*Because of multiple hypotheses testing, a *p* value ≤ 0.01 determined statistical significance

*SD* standard deviation, *CDI Clostridium difficile* infection, *COPD* chronic obstructive pulmonary disease, *WBC* white blood cell, *Hg* hemoglobin, *BUN* blood urea nitrogen

Admission laboratory values were similar and both groups presented with marked leukocytosis, impaired renal function, and hypoalbuminemia reflective of the severity of their infection (Table 2).

Almost all of the patients had received additional antibiotics within 8 weeks of CDI diagnosis, with the most common exposures to penicillins, cephalosporins and quinolones. There were no differences noted in the predisposing antibiotic classes between the two groups.

### Hospital course and outcomes

Table 3 presents the hospital course and outcomes of the 127 patients with moderate to severe CDI. The patients who received intracolonic vancomycin had a longer period of hospitalization compared with the patients who did not receive intracolonic vancomycin, (39.1 ± 30.6 days vs. 27.1 ± 33.3 days), although this difference was not statistically significant (*p* = 0.09). There was an increased incidence of recent VRE and MRSA infections or colonizations in the group that received intracolonic vancomycin compared to the group who received standard treatment without intracolonic vancomycin, *p* = 0.005, *p* = 0.007 respectively (Table 3).

**Table 3:** Hospital course and outcomes of *Clostridium difficile* infected patients who were treated with therapy including adjunctive intracolonic vancomycin compared to those who received therapy without intracolonic vancomycin

	Therapy with adjunctive intracolonic vancomycin No. (%) or average ± s.d.	Therapy without intracolonic vancomycin No. (%) or average ± s.d.	*P* value*
Total Number	26 (20.5 %)	101 (79.5 %)	
Average Severity Score Index (range 0–9)	5.69 ± 1.4	5.02 ± 1.0	0.03
Length of hospital stay (days): average + SD	39.1 ± 30.6	27.1 ± 33.3	0.09
Death	10 (38.5)	39 (38.6 %)	1.00
Oral vancomycin	21 (80.8 %)	44 (43.6 %)	0.0008
Oral metronidazole	13 (50.0 %)	76 (75.2 %)	0.012
IV metronidazole	25 (96.2 %)	45 (44.6 %)	<0.0001
Admission to or transfer to the ICU	24 (92.3 %)	70 (69.3 %)	0.022
Fever (>101 ° F)	10 (38.5 %)	37 (33.7 %)	0.86
Ileus	12 (46.2 %)	29 (28.7 %)	0.10
Toxic megacolon	9 (34.6 %)	4 (4.0 %)	<0.0001
Colectomy	4 (15.4 %)	1 (1.0 %)	0.006
Recent VRE infection and/or colonization	7 (26.9 %)	6 (5.9 %)	0.005
Recent MRSA infection and/or colonization	10 (38.5 %)	14 (13.7 %)	0.007

*Because of multiple hypotheses testing, a *p* value ≤ 0.01 determined statistical significance

*SD* standard deviation, *IV* intravenous, *ICU* intensive care unit, *VRE* vancomycin resistant enterococcus, *MRSA* methicillin resistant *Staphylococcus aureus*

More patients in the intracolonic vancomycin group also received IV metronidazole and oral vancomycin, while the majority of patients in the non-intracolonic vancomycin treatment group received oral metronidazole (Table 3). The doses of intracolonic vancomycin ranged from 0.25g every 24 h to 1g every 6 h, with the most common dose administered being 500 mg every 6 h (16 patients). This variation in doses was similar to other large case series reported in the medical literature (Table 4). In our study, the duration of intracolonic vancomycin was quite variable ranging from 1 to 34 days with a median of 7 days and an interquartile range of 2 – 10 days.

**Table 4:** Large case series reporting *Clostridium difficile* infections (CDI) treated with intracolonic vancomycin per rectum

Reference	Number of patients	Hospital setting	Co-morbidities and outcomes	Intracolonic vancomycin dosing regimens per rectum
Current Series	26 patients over 10 years: 2003 - 2012	Single VA center, Loma Linda, CA	Median age = 69 years Severity Score Index (range 0–9) = 5.2 suggestive of “moderate” CDI Mean WBC = 27 k 16 patients survived, 10 died	Variable doses ranging from 0.25g q24 h to 1g q6h – most common dose 500 mg q6h
Saffouri Gastroenterology 2014 [11]	17 patients over 7 years: 2005–2012.	Single tertiary care teaching hospital, Rochester, MN	Median age = 70 years “Severe” CDI (WBC > 15 k or serum creatinine >1.5x baseline) or “Severe-complicated” CDI (i.e., shock, hypotension, ileus., toxic megacolon or ICU admission) 16 patients survived, 1 died	Variable doses ranging from 0.25g q6 to 1g q6 – most common dose 500 mg q6h
Kim Surg Infect 2013 [10]	47 patients over 2.75 years: 2007 - 2009	Single inner-city hospital, Bronx, NY	Mean age = 65 years “Severe” CDI (any of the following: WBC > 15 k, abdominal tenderness, acidosis) Mean WBC = 27 k 37 patients survived, 10 died	All received 1 g q6h
Apiusarnthanarkak CID 2002 [6]	9 cases over 3 years over 3.5 years: 1998 - 2001	Single tertiary care teaching hospital, St. Louis, MO	Median age = 65 years Mean APACHE II score = 18 (range 12–26) Median WBC = 24 k 8 patients survived, 1 died	Variable doses ranging from 0.5g q4h to 1 g q12h – most common dose 1g q12h
Shetler Surg Endosc 2001 [9]	8 cases	Single VA center, Palo Alto, CA	Mean age = 70 years “Severe” CDI all with ileus and toxic megacolon Median WBC = 24 k 3 patients survived, 4 died	All received 0.25g q6h

## Discussion

We present a retrospective study of 127 patients describing the utility of treating severe CDI using intracolonic vancomycin. To our knowledge this is the second largest case series published to date [6, 9–11] (Table 4).

In this study of patients with moderate to severe CDI, those treated with intracolonic vancomycin were critically ill since more than 90 % were admitted into the ICU and the average length of hospital stay was almost 40 days. This group had a higher incidence of toxic megacolon, which is a serious and life-threatening condition associated with a high mortality rate and systemic toxicity [16, 17]. Additionally this group underwent a greater number of colectomies, a therapy that is often reserved for severe refractory disease [17]. Despite having a greater number of *Clostridium difficile-*associated severe complications and a higher severity score (5.69 vs 5.02), the group that received adjunctive intracolonic vancomycin maintained a similar mortality rate as the group that received standard treatment without intracolonic vancomycin (38.5 vs 38.6 %). The severity score index that had been previously derived and validated in a VA population would have predicted a mortality closer to 50 % [13, 14]. This may suggest a protective role of intracolonic vancomycin.

In our study, patients treated with intracolonic vancomycin were also more likely to be infected or colonized with MRSA and VRE. The predominance of VRE and MRSA in the group that received intracolonic vancomycin may be demonstrative of excessive antibiotic use predisposing to severe CDI [18]. Antibiotic overuse is a major cause of severe CDI [18, 19]. Non-essential antibiotics can eliminate protective colonic flora and predispose to colonization and infection by virulent strains of *Clostridium difficile* [20]. VRE and MRSA are established markers of antimicrobial overuse and patients diagnosed with VRE are at greater risk for developing recurrent CDI [21]. Another explanation for our observation is that CDI has been shown to be a common coinfection in patients diagnosed with VRE secondary to the use vancomycin and metronidazole and prior use of metronidazole [22]. There have been a number of other hypotheses for this relationship, such as the sharing of common risk factors between the two groups [23], treatment with broad spectrum antibiotics for VRE, and use of vancomycin for the treatment of CDI.

Current practice guidelines for the initial management of CDI recommend stratifying patients into mild, moderate and severe CDI [1–3, 24]. However, no single classification has been validated in a broad population or achieved national acceptance for this purpose [25, 26]. We chose to use the severity score index developed by Velazquez-Gomez [14] and validated by Toro [13] because these studies were done in a VA population similar to ours. However, this index would have predicted an approximate 50 % and 100 % mortality in moderate and severe CDI groups respectively, which is in contrast to the 39 % observed for both groups in our study. One explanation for this discrepancy is that the original prediction rule may not be applicable to other populations since it was derived from a small sample of 101 patients and validated in another small sample of 54 patients, both from the same single institution [27]. Another possibility is that because the intracolonic vancomycin group was critically ill with more comorbidities, their physicians were more likely to prescribe intracolonic vancomycin secondary to non-response from oral antibiotics. This confounding by indication is a well known bias of retrospective studies.

Current guidelines vary in their recommendations for the initial treatment of severe CDI and the role of intracolonic vancomycin. The Society for Healthcare Epidemiology of America (SHEA) and Infectious Diseases Society of America (IDSA) [2] recommend, besides oral vancomycin and IV metronidazole, “if complete ileus *consider* (italics added by the authors) adding rectal installation of vancomycin.” Gastrointestinal guidelines [1] recommend for CDI patients complicated with ileus or toxic colitis and/or significant abdominal distention, the “treatment of choice” is oral and per rectum vancomycin plus IV metronidazole. The European Society of Clinical Microbiology and Infectious Diseases [3] recommend one of 3 regimens for “severe, and/or complicated or refractory CDI”: IV metronidazole plus per rectum vancomycin or IV metronidazole plus oral vancomycin or IV tigecycline. The use of rectal vancomycin is based on low quality evidence, mostly case series and expert opinion [1–3]. In our series, many of the cases occurred before the publication of the current guidelines. As with most other case series described in the literature (see Table 4), the dosage of intracolonic vancomycin varied from 0.25 to 1g every 6 h with the most common dosage being 500 mg every 6 h. The latter dose and frequency reflect current practice guidelines recommendations, although this is based on low quality evidence [1, 3]. The duration of use in our study was also quite variable ranging from 1 to 34 days with a median of 7 days. The authors were unable to find the duration of intracolonic vancomycin use in most other published case series listed in Table 4. Current guidelines also vary with their recommendations for duration of use ranging from 10 days [3] to “until the patient improves” [1], but in both situations this is also based on low quality evidence.

Finally, other potential therapies for CDI not used in our case series include the use of intravenous immunoglobulin (IVIG) and Fecal Microbiota Transplantation (FMT). In recent years there has been a renewed focus on the use of Fecal Microbiota Transplantation (FMT) for the treatment of recurrent CDI. The current data on FMT for the treatment of severe CDI is limited only to case reports or small case series [28, 29]. It remains to be seen how FMT will fit into the treatment of severe CDI.

Compared to other case series, the strengths of our study include the consecutive identification of all patients with CDI based on our hospital’s infection control surveillance during the time period studied, systematic collection of information from charts using a standardized form, application of a single severity index that stratified patients into comparable risk groups, and complete follow-up to at least 90 days after the diagnosis of CDI was made to record complications and outcomes.

The limitations of our study include those inherent to most retrospective studies including selection bias, recall bias, and confounding by indication. The population studied comes from a single institution, focusing on a veteran population, which may also limit this study’s application to more diverse populations. As noted in Table 3, although the severity score indices between the two groups were not statistically significant, the baseline therapy between the two groups differed significantly. The non-intracolonic vancomycin group received more oral metronidazole compared with the intracolonic vancomycin group, which received more IV metrondizaole and oral vancomycin. Since this was a retrospective study over 10 years, the “standard” of care evolved and primary care providers varied in their treatment approaches which has the potential to confound the study results. As noted in Table 4, the clinical indications for intracolonic vancomycin, doses and duration of uses, and outcomes vary significantly in this study and among other reported cases series in the literature. Data differentiating PCR-positive, toxin-negative patients from PCR-positive, toxin-positive patients were not collected and the specific strain of CDI for each case was also not available. Finally, the standardized information form recorded only all-cause 90 day mortality without a specified cause of death.

## Conclusion

Intracolonic vancomycin is a promising adjunctive therapy for patients with severe CDI, which carries a high mortality rate. In our retrospective study, despite having a higher incidence of *Clostridium difficile*-associated severe complications and being more critically ill, the group that received adjunctive intracolonic vancomycin demonstrated a similar mortality rate compared to the group that did not receive this therapy. While intriguing to hypothesize that intracolonic vancomycin may reduce the morbidity and mortality of severe CDI, only a prospective randomized controlled trial can determine the true causality of the effectiveness of this potentially important adjunctive therapy.

## Abbreviations

CDI, *Clostridium difficile* infection; EIA, enzyme immunoassay; FMT, fecal microbiota transplantation; GDH, glutamate dehydrogenase; ICU, intensive care unit; IDSA, Infectious Diseases Society of America; PCR, polymerase chain reaction; SHEA, Society for Healthcare Epidemiology of America; VA, Veterans Affairs; VRE, vancomycin resistant enterococcus
